# An Investigation of the Influence of PEG 400 and PEG-6-Caprylic/Capric Glycerides on Dermal Delivery of Niacinamide

**DOI:** 10.3390/polym12122907

**Published:** 2020-12-04

**Authors:** Yanling Zhang, Majella E. Lane, David J. Moore

**Affiliations:** 1 Department of Pharmaceutics, UCL School of Pharmacy, 29-39 Brunswick Square, London WC1N 1AX, UK; m.lane@ucl.ac.uk; 2 Tioga Research, Inc., Edinburgh EH31 2BA, UK; dmoore@tiogaresearch.com

**Keywords:** niacinamide, polyethene glycol (PEG) 400, solvent, dermal delivery, finite dose, porcine skin

## Abstract

Polyethylene glycols (PEGs) and PEG derivatives are used in a range of cosmetic and pharmaceutical products. However, few studies have investigated the influence of PEGs and their related derivatives on skin permeation, especially when combined with other solvents. Previously, we reported niacinamide (NIA) skin permeation from a range of neat solvents including propylene glycol (PG), Transcutol^®^ P (TC), dimethyl isosorbide (DMI), PEG 400 and PEG 600. In the present work, binary and ternary systems composed of PEGs or PEG derivatives combined with other solvents were investigated for skin delivery of NIA. In vitro finite dose studies were conducted (5 μL/cm^2^) in porcine skin over 24 h. Higher skin permeation of NIA was observed for all vehicles compared to PEG 400. However, overall permeation for the binary and ternary systems was comparatively low compared with results for PG, TC and DMI. Interestingly, values for percentage skin retention of NIA for PEG 400:DMI and PEG 400:TC were significantly higher than values for DMI, TC and PG (*p* < 0.05). The findings suggest that PEG 400 may be a useful component of formulations for the delivery of actives to the skin rather than through the skin. Future studies will expand the range of vehicles investigated and also look at skin absorption and residence time of PEG 400 compared to other solvents.

## 1. Introduction

The skin is the largest organ of the human body and serves as a unique interface between humans and the environment. This membrane is a formidable barrier, preventing the egress of water, the ingress of toxins and offers protection against ultraviolet (UV) radiation [1,2]. However, skin also serves as a route for the administration of therapeutic molecules for both local and systemic effects [3]. The primary challenge in the skin penetration process is the passage of molecules through the outermost layer, the stratum corneum (SC). The SC consists of eight to sixteen layers of [4] keratinized corneocytes embedded in a lipid domain. To improve the efficacy of topical and transdermal delivery, various formulation components have been investigated for their potential to facilitate permeation of actives through the SC.

Polyethylene glycols (PEGs) are synthetic polymers of condensed ethylene oxide (EO) and water [5]. These polymers and their derivatives have numerous applications in the food industry and the pharmaceutical and biomedical fields [6,7]. With reference to skin formulations, PEGs and their derivatives are primarily used as solvents, surfactants or stabilizers. Surprisingly, few publications have examined the mechanistic effects of PEGs on the permeation of actives. Sarpotdar and colleagues investigated the effect of PEG 400/water mixtures on the penetration of oxaprozin and guanabenz in cadaver skin using in vitro diffusion cell studies [8]. The flux values of both drugs were found to drop linearly as the concentration of PEG 400 increased in the formulations. Suwanpidokkul et al. compared the penetration of zidovudine in porcine skin from PEG 400/water vehicles with ethanol/water, isopropyl alcohol/water and ethanol/isopropyl myristate (IPM) vehicles. Flux values for the PEG 400/water vehicle were comparable to those for ethanol/water and isopropanol/water, namely 15.52 ± 0.60, 9.96 ± 0.63 and 10.35 ± 0.67 μg/cm^2^/h, respectively. However, both of these studies used infinite dose conditions; therefore, it is difficult to extrapolate the findings for typical “in use” conditions for topical preparations. Aside from the very limited evaluation of PEGs on the skin delivery of actives, comparative studies have not been reported for PEG derivatives, either as neat solvents or as components of simple binary or ternary systems.

Niacinamide (NIA) is the water-soluble form of vitamin B3 and the amide form of nicotinic acid [9]. In 1976, Comaish et al. [10] first reported NIA as an efficient treatment for pellagra, a vitamin deficiency disease with dermatitis symptoms. Since then, topical application of NIA has been shown to improve various skin conditions in humans. The beneficial effects include skin barrier enhancement [9], reduction of hyperpigmentation [11], anti-inflammation [12] and prevention of UV-induced immunosuppression [13]. Favorable compatibility with the active is a crucial criterion when selecting formulation components for dermal delivery of NIA. All solvents investigated in this study have good safety profiles and widespread applications in pharmaceutical and consumer products. Propylene glycol (PG) is the most commonly used glycol in topical and transdermal formulations [2]. Data from both clinical and nonclinical studies support the use of PG as a nontoxic and well-tolerated material [14]. Transcutol^®^ P (TC) and dimethyl isosorbide (DMI) are commonly used vehicles in skin preparations with GRAS status [15,16]. PEG 400 is an FDA approved polymer for use in drug delivery systems because of its safety and tolerance when administered to the body by different routes [17]. PEG-6-CCG was selected as it is currently the most widely used PEG alkyl glyceride derivate in personal care products [18]. According to the 2014 FDA Voluntary Cosmetic Registration Program (VCRP) data, PEG-6-CCG was used in 548 formulations, and the CIR Expert Panel confirmed the GRAS status of the compound [18]. Previously, we reported the dermal delivery of NIA using porcine skin in vitro, from a range of simple solvents for both infinite and finite dose conditions [19]. The vehicles examined included propylene glycol (PG), Transcutol^®^ P (TC), dimethyl isosorbide (DMI), t-butyl alcohol (T-BA) and PEGs 400 and 600. For finite dose studies, the percentage permeation of NIA was ranked as follows: T-BA> DMI > TC > PG; no NIA permeation was observed for PEGs 400 or 600. Corresponding mass balance studies confirmed that NIA was largely deposited on the skin surface with the PEG vehicles. We hypothesized that by combining PEG 400 with neat solvents effective in promoting NIA skin permeation, NIA skin penetration might be enhanced when compared with PEG 400. Thus, the aims of the present study were to (i) design binary and ternary systems of PEG 400 and/or the PEG derivative PEG-6-caprylic/capric glycerides (PEG-6-CCG) and (ii) investigate any synergistic effects of these systems on the permeation of NIA in porcine skin, compared with the neat solvents studied previously.

## 2. Materials and Methods

### 2.1. Materials

NIA, PG, PEG 400, high-performance liquid chromatography (HPLC) grade water and methanol were purchased from Sigma-Aldrich, Dorset, UK. TC was a gift from Gattefossé, St. Priest, France, and Croda Ltd., Goole, UK, supplied the DMI. PEG-6-caprylic/capric glycerides (PEG-6-CCG) was a gift from Avon, Suffern, NY, USA. Phosphate buffered saline (PBS) tablets (pH 7.3 ± 0.2 at 25 °C) were purchased from Oxoid, Cheshire, UK.

### 2.2. Determination of Solubility, Solubility Parameters and HPLC Analytical Method

The method for determining solubility of NIA in PG, TC, DMI, PEG 400, PEG 600 at 32 ± 1 °C has been reported previously [19]. The solubility of NIA in PEG-6-CCG under the same conditions was evaluated by adding an excess amount of NIA to a known volume of PEG-6-CCG. The mixture was continuously stirred with a Teflon coated magnetic bar for 48 h at 32 ± 1 °C. After 48 h, samples were centrifuged (13,200 rpm) for 15 min, and the supernatant was collected and diluted with methanol–water (50:50). All solubility values were reported as % *w/v*.

The van Krevelen Hoftyzer solubility parameters (δ) for NIA and solvent systems were calculated using Molecular Modeling Pro^®^ (ChemSW Inc., Fairfield, CA, USA). For the binary and ternary systems, as shown in Equations (1) and (2), the solubility parameters (δBinary/Ternary) were calculated according to the respective mole fractions (Φ1, Φ2 …) for the solvents and their respective solubility parameters (δ1, δ2 …) [20].

(1) $$\delta_{Binary}=\frac{\delta_{1}\Phi_{1}+\delta_{2}\Phi_{2}}{\Phi_{1}+\Phi_{2}}\;$$

(2) $$\delta_{Ternary}=\frac{\delta_{1}\Phi_{1}+\delta_{2}\Phi_{2}+\delta_{3}\Phi_{3}}{\Phi_{1}+\Phi_{2}+\Phi_{3}}\;$$

Samples of NIA were analyzed using HPLC [19]. A Kinetex 5 μm Phenyl-Hexyl 250 × 4.6 mm reverse phase column (Phenomenex, Macclesfield, UK) packed with a SecurityGuard™ cartridge (Phenomenex, UK) was used to achieve separation. The column temperature was set at 30 °C. The mobile phase was water–methanol (80:20) with a flow rate of 1 mL/min. The injection volume was set to 10 μL, and the UV detection wavelength was 263 nm. The HPLC method was validated previously according to ICH guidelines (2005) [21], including accuracy, precision, detection limit, quantitation limit, linearity and robustness.

### 2.3. Dynamic Vapor Sorption (DVS) Studies

To study the evaporation or hydration of NIA solutions, a Q5000 SA sorption analyzer (TA Instruments, New Castle, DE, USA) was used to examine the mass variation over 24 h (1440 min) following the procedure reported by Iliopoulos et al. [22]. Nitrogen was used as the carrier gas (200 mL/min). To monitor mass differences, 15 mm quartz glass pans connected to a microbalance accurate to 0.00001 mg were employed. Then, 5 μL solutions of 5% NIA (*w/v*) were added to the pans, and data were recorded using a Universal Analysis 2000 (TA Instrument, New Castle, DE, USA). Temperature and relative humidity (RH) were maintained over the course of the studies at 32 ± 1 °C and 50 ± 2% RH, respectively.

### 2.4. Permeation Studies and Mass Balance Studies

Binary and ternary solvent systems were prepared for the in vitro permeation studies, namely PEG 400:TC (50:50), PEG 400:PG (50:50), PEG 400:DMI (50:50), PEG 400:PEG-6-CCG (50:50) and PEG 400:PG:DMI (50:25:25). The concentration of NIA typically used in personal care products ranges from 2% to 5% [23]. To be consistent with previous studies, a concentration of 5% (*w/v*) NIA was therefore selected in the present work.

In vitro permeation studies were performed using Franz diffusion cells [24,25]. Full thickness porcine ear skin has been proposed as an appropriate surrogate for human skin [26]. The in vitro skin permeation procedure was designed based on current Organisation for Economic Co-operation and Development (OECD) guidelines [27]. Porcine tissue was obtained from a local abattoir and prepared on the same day of collection. The skin was surgically separated from the cartilage following a standardized procedure [28]. After preparation, the porcine skin was mounted on aluminum foil and stored at −20 °C until required. Skin integrity was confirmed by assessing electrical resistance before experiments [18,29]. A 2.5 mL degassed freshly prepared PBS solution (pH 7.30 ± 0.20) was used as the receptor medium. To mimic clinically relevant conditions, 5 μL of the NIA solution was applied over a diffusion area of ~1 cm^2^ after the skin temperature had equilibrated to 32 ± 1 °C. The effective diffusion area was measured accurately using a Vernier Caliper (Fisher Scientist, Loughborough, UK). At each sampling point, 200 μL of the receptor medium was withdrawn and refilled with an equal volume of fresh PBS solution for up to 24 h. At the end of the permeation studies, after removing the receptor medium, mass balance studies were performed to determine the total distribution of NIA following a validated procedure [24]. The details of the mass balance method are provided in the Supplementary Materials section of this paper.

### 2.5. Statistical Analysis

All data are presented as the mean ± standard deviation (SD). The statistical analysis was performed using SPSS^®^ Statistics version 24 (IBM, Feltham, UK). The normality of data was examined using the Shapiro–Wilk test, and the homogeneity of variance was examined using Levene’s test. An independent t-test or one-way ANOVA with a post hoc Tukey test was performed for data that met the assumption of normality and homogeneity of variance. The Kruskal–Wallis H test was used for nonparametric data. A *p*-value lower than 0.05 (*p* < 0.05) was considered a significant difference.

## 3. Results and Discussion

### 3.1. Solubility Studies

The solubility parameters of the various vehicles investigated, and corresponding NIA solubility values, are shown in Table 1. The solubility parameters for PEG-6-CCG and the binary systems composed of PEG-6-CCG were not calculated, as this solvent is a mixture of polyethylene glycol derivatives of a range of caprylic/capric glyceride acids [30]. The solubility parameter of NIA was reported as 13.9 (cal/cm^3^)^1/2^, and the solubility values for NIA in DMI, TC, PEG 400 and PG were previously reported [19]. The NIA solubility value for PEG 400:PG (50:50) was 25.6 ± 1.1% (*w/v*). This value was significantly higher (Table 1) than the results obtained for PEG 400:PEG-6-CCG (50:50), PEG 400:DMI (50:50), PEG 400:TC (50:50) and PEG 400:PG:DMI (50:25:25), (*p* < 0.05).

The NIA solubility values in neat PEG-6-CCG and the binary and ternary systems evaluated here have not previously been reported. The solubility of NIA in PEG-6-CCG was found to be 5.6 ± 0.3%, which was lower than the solubility in neat DMI, PEG 400, TC and PG (*p* < 0.05). For the various binary solvent systems, the NIA solubility ranged from 15.0 to 25.6%. Compared to neat DMI, NIA had a higher solubility in the binary PEG 400:DMI system, determined as 17.6 ± 1.2% (*p* < 0.05). Furthermore, the solubility of NIA in PEG 400:TC (50:50), 23.5 ± 0.1%, was significantly greater compared to the solubility in neat PEG 400 and TC (*p* < 0.05). NIA solubility values in PEG 400:PEG-6-CCG (50:50) and PG:PEG-6-CCG (50:50) were determined as 15.0 ± 1.0 and 25.4 ± 0.1%, respectively. Both values were significantly higher than the result obtained for NIA solubility in neat PEG-6-CCG (*p* < 0.05).

### 3.2. DVS Studies

**Figure 1:**
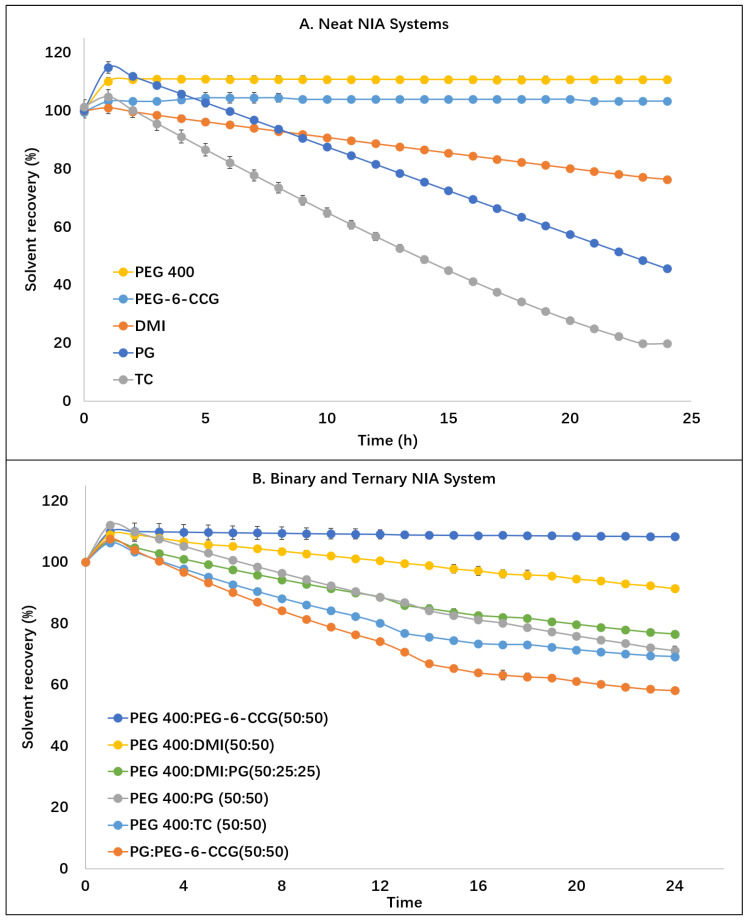
Dynamic Vapor Sorption (DVS) studies of NIA (5%) in propylene glycol (PG), Transcutol® P (TC), dimethyl isosorbide (DMI) and PEG400 and PEG-6-CCG solutions (**A**) and PEG:PEG-6-CCG (50:50), PEG400:DMI (50:50), PEG400:PG (50:50), PEG400:TC (50:50), PG:PEG-6-CCG and PEG400:PG:DMI (50:25:25) (**B**) over 24 h at 32 ± 1 °C and 50 ± 2% RH (n = 3, mean ± SD).

Figure 1 shows the DVS results for the various NIA systems over a 24 h period at 32 ± 1 °C and 50 ± 2% RH. Similar trends were observed for NIA in PG, TC and DMI. The initial increase in mass for NIA in PG, TC and DMI reflects the hygroscopic nature of these solvents. After 1 h, the mass of the PG, TC and DMI solutions started to decrease as a result of solvent evaporation. At 24 h, 44.6 ± 0.02, 17.6 ± 0.5 and 76.3 ± 0.1% of the applied weights of the PG, TC and DMI solutions were recovered, respectively. These results are similar to the findings for the evaporation of neat PG and TC reported by Haque et al. [31], and the mass loss for the DMI solution is also consistent with the findings of Iliopoulos et al. [22]. For PEG 400 and PEG-6-CCG, the mass increase likely reflects water retention by these solvents with 110.7 ± 1.2 and 103.3 ± 0.4% recovered at 24 h, respectively. Statistical differences were evident between the percentage recovery values determined at 24 h for the PEG 400 and the PEG-6-CCG formulations (*p* < 0.05). To our knowledge, DVS has not been previously used to characterize the behavior of PEG-6-CCG under controlled conditions of temperature and humidity.

Figure 1B shows the DVS results for the binary and ternary systems of NIA over 24 h. As for the results observed for neat PEG 400 and PEG-6-CCG, an initial increase in weight was evident for all binary and ternary systems, again reflecting the hygroscopic nature of PEG 400 and PEG-6-CCG. For the ternary PEG 400:PG:DMI system, 76.5 ± 1.0% of the initial applied mass was recovered at 24 h. This value was significantly higher than the results for neat PG (*p* < 0.05). As expected, after the initial increase in weight at the start of the experiment, no further mass changes were observed for the PEG 400:PEG-6-CCG NIA system. For the remaining binary systems composed of PG, TC or DMI, the mass decreased after 1 h, resulting from evaporation of these solvents. At 24 h, 58.1 ± 0.8 and 71.1 ± 1.5% of the applied amounts of PG:PEG-6-CCG (50:50) and PEG 400:PG (50:50) NIA solutions were recovered, respectively. These values were higher than the corresponding results for neat PG (*p* < 0.05). At 24 h, 69.1 ± 1.0 and 91.4 ± 0.8% of the applied PEG 400:TC (50:50) and PEG 400:DMI (50:50) mass were recovered, respectively, and these values were also significantly higher than the values for neat TC or DMI (*p* < 0.05).

### 3.3. In Vitro Permeation Studies

**Figure 2:**
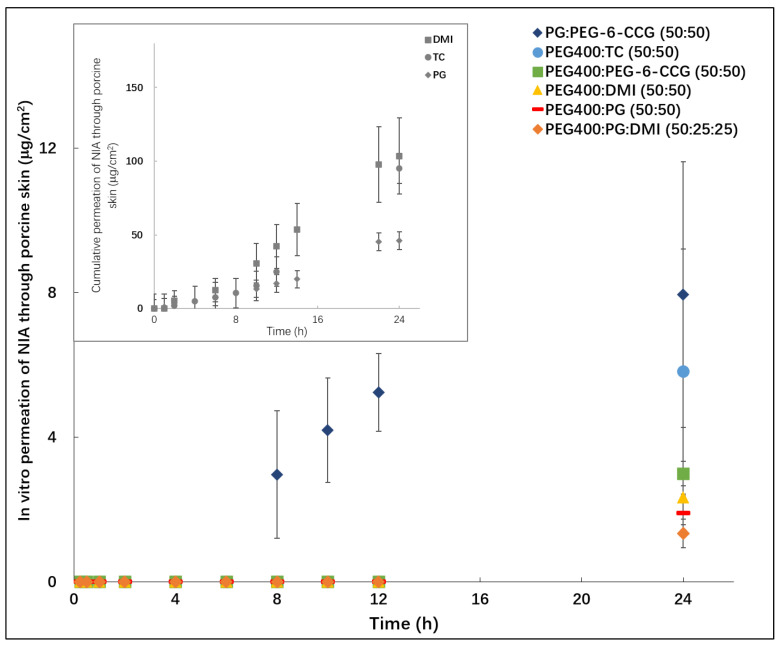
In vitro permeation of NIA from binary and ternary solvent systems under finite dose conditions (5 μL/cm^2^) (n = 4, mean ± SD). The permeation profiles of NIA from single solvents (DMI, TC and PG) were adopted from Zhang et al. [19].

The permeation profiles for all vehicles are shown in Figure 2. All experiments were performed using Franz diffusion cells and porcine skin under finite dose conditions. For the binary PG:PEG-6-CCG (50:50) system, permeation of NIA was not evident until the 8 h sampling point; for all other systems, NIA was only detected at the end of the permeation study. At 24 h, NIA permeation from PG:PEG-6-CCG (50:50) and PEG 400:TC (50:50) was determined as 8.0 ± 3.7 and 5.8 ± 3.4 μg/cm^2^. The corresponding values for PEG 400:PEG-6-CCG (50:50), PEG 400:DMI (50:50), PEG 400:PG (50:50) and PEG 400:PG:DMI (50:25:25) were 3.0 ± 0.3, 2.3 ± 0.7, 1.9 ± 0.5 and 1.3 ± 0.4 μg/cm^2^, respectively. Statistical analysis confirmed a significant difference between NIA permeation for PG:PEG-6-CCG and PEG 400:PG:DMI (*p* < 0.05). As reported previously, the corresponding cumulative permeation values of NIA for neat PG, DMI and TC solutions were 46.0, 103.6 and 95.1 μg/cm^2^ at 24 h, respectively; no permeation of NIA was observed for neat PEG 400 [19].

**Figure 3:**
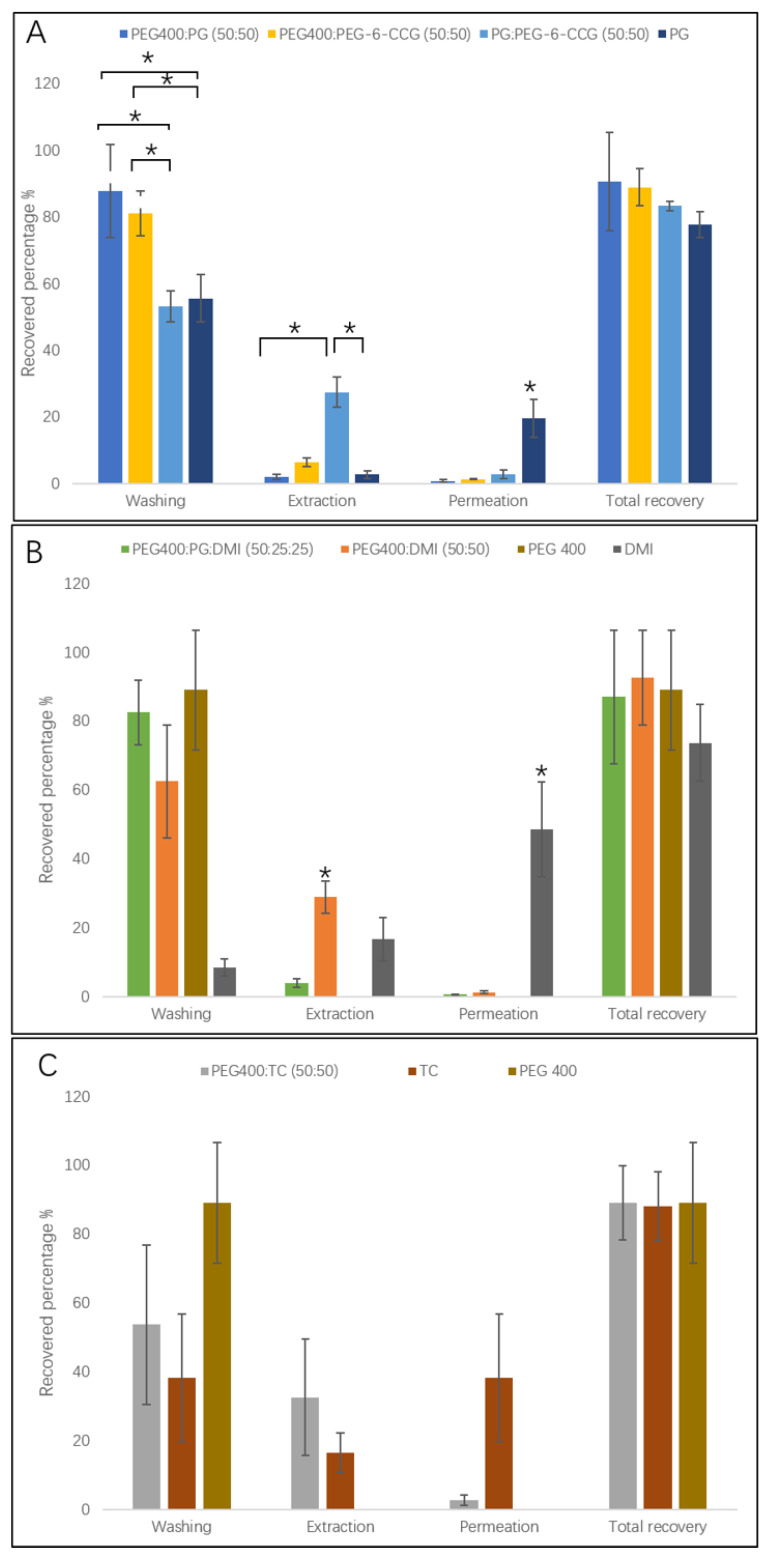
Mass balance results of the applied NIA from binary and ternary systems after 24 h permeation studies (n = 4, mean ± SD). (**A**) summarizes the mass balance results from PEG400:PG (50:50), PEG400:PEG-6-CCG (50:50), PG:PEG-6-CCG (50:50) and neat PG; (**B**) summarizes the results from PEG400:PG:DMI (50:25:25), PEG400:DMI (50:50), PEG400 and DMI; (**C**) summarizes the results from PEG400:TC (50::50), TC and PEG400. The mass balance results of NIA from neat TC, PG, PEG 400 and DMI were adopted from Zhang et al. [19]. Statistical analysis was highlighted in the figure (*, *p* < 0.05).

The results of the mass balance studies are summarized in Figure 3A–C. The corresponding percentage values determined for neat PEG 400, PG, TC and DMI, reported previously [19], are also included in the figures for comparison. For the binary and ternary vehicles, the values for total recovery of NIA were within the recommended ranges as published in the Scientific Committee on Consumer Safety (SCCS) guidelines for dermal absorption studies (85–115%) [32]. For the binary and ternary systems, 0.6 to 2.7% of the applied NIA penetrated through the skin membrane after 24 h permeation. Lower NIA permeation percentage values were still evident for the binary/ternary systems compared to neat PG, TC and DMI (*p* < 0.05).

The percentage of the applied dose extracted from the skin membrane ranged from 2 to 33% for all assessed binary vehicles. The percentage retention of NIA was 32.6 ± 16.9% for PEG 400:TC (50:50), followed by PEG 400:DMI (50:50) and PG:PEG-6-CCG (50:50), with corresponding values of 28.9 ± 4.7 and 27.4 ± 4.6%, respectively. In comparison to neat PG, a higher NIA skin retention was evident for PG:PEG-6-CCG (50:50) (*p* < 0.05) (Figure 3A). Previously, we reported skin retention of NIA as 16.7 ± 6.3% for neat DMI [19]. Hence, the binary PEG 400-DMI system also significantly improved NIA skin retention compared with neat DMI (*p* < 0.05) (Figure 3B). The percentage of NIA extracted from skin for PEG 400:TC was 32.6 ± 16.9%, but no significant difference was evident in comparison to neat TC (16.4 ± 5.8%, *p* > 0.05) (Figure 3C).

PG has been used in a range of topical and transdermal formulations [2]. Hoelgaard et al. [33] reported a “carrier-solvent” effect for PG enhancement of metronidazole permeation. Haque et al. [31] investigated the skin delivery of anthramycin from a PG solution and noted that anthramycin appeared to “track” the permeation of PG. The authors suggested that PG might enhance skin permeability by increasing the partition or solubility of the drug in skin. Recently, Kung et al. [34] investigated the skin penetration of methadone from binary systems composed of PG and other solvents. Increased permeation of methadone corresponded to high skin uptake of PG. These works suggest that PG may interact with skin lipids and influence the barrier function of the SC. The results reported here indicate that combining PG with PEG 400 does not promote enhanced permeation or skin retention compared with PG. However, the combination of PG with PEG-6-CCG improved the percent skin retention of NIA compared to PG alone (*p* < 0.05). NIA skin retention was comparable for PEG 400:PG (*p* > 0.05) and neat PG (*p* < 0.05). As the solubility values for NIA in PG, PEG 400:PG and PG:PEG-6-CCG are comparable (Table 1), other properties of the solvents appear to influence the skin permeation of NIA.

Reports in the literature suggest that TC might increase percutaneous absorption by changing the solubility of drugs in the skin rather than disrupting skin lipids [35]. The comparatively rapid skin permeation of TC compared with PG, 1,3-butanediol and dipropylene glycol was reported by Haque et al. [31]. The solubility of NIA in TC was previously reported as 8.0 ± 0.6% [19]. In the present work of combining PEG 400 with TC, significantly higher solubility values for NIA were evident compared with those for neat TC or PEG 400 (*p* < 0.05). Combining DMI with PEG 400 resulted in a two-fold increase in NIA solubility. As for the PG results, high solubility in the binary DMI and TC vehicles did not promote skin permeation of NIA compared to TC or DMI alone.

## 4. Conclusions

PEGs and their derivatives are commonly used in topical and transdermal formulations. Previously, we reported no permeation or skin retention of NIA from a neat PEG 400 solution in porcine skin. The present work examined NIA skin delivery from vehicles composed of PEG 400 and the PEG derivative PEG-6-CCG. Permeation of NIA was increased for the PEG 400 binary systems compared to PEG 400 alone, but overall, very low permeation of NIA was observed. On the other hand, high skin retention was observed for these vehicles when compared with the neat solvents investigated. Depending on the active of interest, skin retention rather than permeation may be more desirable. An assessment of the residence time of PEGs on and in the skin should also allow for a better understanding of how PEGs can be utilized when targeting actives to the skin. This will be the focus of future work, as well as an investigation of the influence of other PEGs and their derivatives on skin delivery of actives.

## Figures and Tables

**Table 1:** Solubility of niacinamide (NIA) in tested single, binary and ternary solvent systems at 32 ± 1 °C (mean ± SD, n = 3). The solubility parameter of NIA was reported as 13.9 (cal/cm^3^)^1/2^. Statistical analysis is highlighted in the table (*, *p* < 0.05).

Solvent Systems (*v*/*v*)	Solubility Parameter (cal/cm^3^ )^1/2^	Solubility of NIA ((%, g/100 mL)
PEG-6-CCG	-	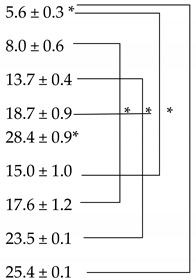
DMI	10.0	
TC	10.6	
PEG 400	11.7	
PG	14.1	
PEG 400:PEG-6-CCG (50:50)	-	
PEG 400:DMI (50:50)	10.9	
PEG 400:TC (50:50)	11.2	
PG:PEG-6-CCG (50:50)	-	
PEG 400:PG (50:50)	12.8	25.6 ± 1.1
PEG400:PG:DMI (50:25:25)	11.9	21.9 ± 0.8

## Supplementary Materials

The following are available online at https://www.mdpi.com/2073-4360/12/12/2907/s1, File S1: The Method of Mass Balance Study.
