# Supplementary material for: An Investigation of the Influence of PEG 400 and PEG-6-Caprylic/Capric Glycerides on Dermal Delivery of Niacinamide
Source: Polymers (Basel). 2020 Dec 4;12(12):2907. doi: 10.3390/polym12122907 (PMC7761779; doi:10.3390/polym12122907)
Supplement: Supplementary file 1 [file polymers-12-02907-s001.pdf]

Procedure for mass balance studies: At the end of the permeation studies, the receptor phase was removed completely, and the mass balance study was conducted. The skin surface was washed three times with 1 mL of methanol-water (50:50) mixture, followed by swabbing with a cotton bud. The washing solutions and cotton buds were collected and placed in 2 mL Eppendorf® tubes (Eppendorf®, UK) with 1 mL of methanol-water (50:50) solution. To extract the quantity of NIA in the skin, the skin membrane was removed and cut into small pieces and placed in 2 mL Eppendorf ® tubes with 1 mL of methanol-water (50:50) solution. All the tubes were placed in an incubating orbital shaker at 32 ± 1°C, at a speed of 800 rpm (VWR International, LLC, USA) overnight. After extraction was completed, the tube was centrifuged at 13,200 rpm (5415 R centrifuge Eppendorf®, UK). The supernatant was collected for analysis. NIA quantification was performed using the validated HPLC method. Samples were diluted where necessary.
